# Immunogenicity of Isogenic IgG in Aggregates and Immune Complexes

**DOI:** 10.1371/journal.pone.0170556

**Published:** 2017-01-23

**Authors:** J. Benjamin St. Clair, Thiago Detanico, Katja Aviszus, Greg A. Kirchenbaum, Merry Christie, John F. Carpenter, Lawrence J. Wysocki

**Affiliations:** 1 Department of Biomedical Research, National Jewish Health, Denver CO, United States of America; 2 Medical Scientist Training Program, University of Colorado School of Medicine, Denver, Colorado, United States of America; 3 Integrated Department of Immunology, National Jewish Health and University of Colorado School of Medicine, Denver, Colorado, United States of America; 4 Department of Pharmaceutical Sciences, University of Colorado Denver, Anschutz Medical Campus, Aurora, Colorado, United States of America; Emory University School of Medicine, UNITED STATES

## Abstract

A paradox in monoclonal antibody (mAb) therapy is that despite the well-documented tolerogenic properties of deaggregated IgG, most therapeutic IgG mAb induce anti-mAb responses. To analyze CD4 T cell reactions against IgG in various physical states, we developed an adoptive transfer model using CD4+ T cells specific for a Vκ region-derived peptide in the hapten-specific IgG mAb 36–71. We found that heat-aggregated or immune complexes (IC) of mAb 36–71 elicited anti-idiotypic (anti-Id) antibodies, while the deaggregated form was tolerogenic. All 3 forms of mAb 36–71 induced proliferation of cognate CD4+ T cells, but the aggregated and immune complex forms drove more division cycles and induced T follicular helper cells (T_FH_) development more effectively than did the deaggregated form. These responses occurred despite no adjuvant and no or only trace levels of endotoxin in the preparations. Physical analyses revealed large differences in micron- and nanometer-sized particles between the aggregated and IC forms. These differences may be functionally relevant, as CD4+ T cell proliferation to aggregated, but not IC mAb 36–71, was nearly ablated upon peritoneal injection of B cell-depleting antibody. Our results imply that, in addition to denatured aggregates, immune complexes formed *in vivo* between therapeutic mAb and their intended targets can be immunogenic.

## Introduction

The widespread administration of therapeutic monoclonal antibodies (mAb) has revealed a paradox in the immune response to immunoglobulin-derived antigens. While the historical literature would suggest that soluble, bivalent IgG is profoundly tolerogenic and suppresses Ig-specific humoral responses, therapeutic mAbs can be immunogenic and commonly elicit anti-Id responses in some percentage of recipients, particularly in populations treated for autoimmune diseases. The clinical ramifications of anti-mAb have varied in individual clinical trials, but meta-analyses have confirmed decreased therapeutic efficacy and increased adverse events such as hypersensitivity reactions [1]. To prevent the development of high affinity antibodies directed against therapeutic mAb, researchers and physicians have adopted a number of strategies, with varying practical and theoretical costs and benefits, many of which target CD4+ T cell responses to Ig-derived peptides [1–4].

This focus on the immunogenicity, or tolerogenicity, of Ig for CD4+ T cells is based upon a historical dichotomy in the literature. Dresser first revealed the tolerogenicity of deaggregated, heterologous gamma globulin in 1961 [5–7]. Chiller, Habicht, and Weigle demonstrated that both mouse T helper cells and B cells could be tolerized by polyclonal, deaggregated human gamma globulin, and that T cell tolerance was both long lived and dominant when thymocytes were adoptively transferred into irradiated animals along with normal bone marrow [8–10]. In contrast, Janeway and Paul reported the augmentation of anti-idiotypic antibody production to a hapten-conjugated antibody if mice received a hapten-targeted antisera [11]. This suggested a potential adjuvant role for immune complexes, however the experiment was complicated by the hapten-conjugation to the targeted antibody which led to low anti-idiotypic production without antisera, a potential consequence of novel T-epitopes, aggregation, or endotoxin [12]. In a more recent study, Reitan and Hannestad found that a pentameric IgM form of a monoclonal Ig without adjuvant or endotoxin was immunogenic, while the IgG form was not, even after multiple injections [13–15]. Finally, inclusion of certain peptides into the structure of IgG renders them tolerogenic for CD4+ T cells and mitigates pathology in a mouse model of autoimmune disease [16–25].

Despite evidence for the tolerogenic properties of IgG, therapeutic IgG mAbs often elicit IgG antibody directed against the infused mAb [26–30]. This occurs even when the therapeutic mAb are encoded by entirely human Ig genes. While the CD4+ T cell repertoire attains self-tolerance to germline Ig V region sequences, somatically generated diversity arising at boundaries of V region genes during B cell development or throughout the entire V region via somatic hypermutation is potentially antigenic [13–15, 31–40]. In cases where it is antigenic, this somatic diversity may provide an avenue of T cell help to any B cell specific for the idiotype of a therapeutic mAb. However, antigenic peptide sequences in Ig alone may be insufficient to elicit a productive anti-Id response, which has led researchers to hypothesize that mAbs are more likely to be immunogenic if they are aggregated during handling, targeted to a cell surface antigen, or engaged in immune complexes [41–49]. Prior studies in experimental models generally assessed CD4+ T cell reactions to IgG under circumstances in which the IgG could not form immune complexes *in vivo*, either because the IgG was polyclonal and nonspecific or because the cognate antigen for a monoclonal IgG was not present [38, 40, 50–65]. And in most of these studies, analyses were limited because they involved wildtype i.e. nontransgenic T cells.

To resolve this divide between immunogenicity and tolerogenicity of IgG, we have sought to investigate the *in vivo* response of a single CD4+ T cell clone to an antigenic IgG mAb in various physical states. Using an adoptive transfer model, we demonstrate that aggregated and complexed Ig without adjuvant are immunogenic and elicit IgG anti-Id antibodies, while monomeric Ig induces a profound state of self-tolerance that subverts an anti-idiotypic response.

This dichotomy between immunogenicity and tolerance is mirrored by differences in the early proliferation of antigen-specific CD4+ T cells and development of T_FH_. Finally, we show that heat-aggregated and complexed Ig, while both immunogenic, have notably different structural profiles and distinct requirements for CD4 T cell activation *in vivo*. Taken as a whole, our data suggest that immune complexes may be a common catalyst for productive activation of CD4+ T cells that drive anti-Id responses against therapeutic IgG targeting soluble antigen, in stark contrast to the tolerogenicity of these mAb in an uncomplexed deaggregated form.

## Materials and Methods

### Mice

Three background strains, A/J, C;129S4-Rag2^tm1.1Flv^ Il2rg^tm1.1Flv^/J (Rag2-/-cγ-/-) and (B6 x A/J)F1 (B6AF1) mice were bred in house. A/J CA30 Tg mice (CA30) have been described previously and were maintained on an A/J κ^-/-^ background in-house [55, 57]. B6.SJL-Ptprc^a^Pepc^b^/BoyJ (B6.SJL) mice were purchased from The Jackson Laboratory (Bar Harbor, ME). All mice were housed in the Biological Resource Center at National Jewish Health (Denver, CO). CA30 mice were bred to B6.SJL (CA30.CD45.1) mice to create congenically marked CA30 T cells for adoptive transfers into B6AF1 mice. Mice used for experiments were generally 8–14 weeks old unless otherwise indicated and included both sexes. The National Jewish Health Institutional Animal Care and Use Committee (IACUC) approved this study and all mice were handled and bred with IACUC approval in accordance with institutional guidelines. None of the animals used in this work became ill or died prior to the experimental endpoint. If animals had exhibited symptoms of severe illness/moribundity, they would have received medical treatment or been humanely euthanized. All animals were euthanized per National Jewish Health IACUC guidelines using humane application of carbon dioxide.

### Generation, purification, and storage of mAbs

MAb 36–71 and mAb 36–65 were produced from ascites grown in C;129S4-Rag2^tm1.1Flv^ Il2rg^tm1.1Flv^/J (Rag2-/-cγ-/-) mice that were injected with the respective hybridomas [66]. After clotting, ascites fluid was centrifuged at 20,000 x g for 30 minutes at 4°C and passed through a 0.22 μm filter (Millipore, Billerica, MA) under sterile conditions. IgG was precipitated on ice with (NH4)2SO4 (45% v/v final), centrifuged and dissolved in phosphate buffered saline (PBS) with 0.01% NaN3. The dissolved precipitate was extensively dialyzed against 10 mM NaPO4 pH 7.9, purified by anion exchange chromatography with a DE52 resin. Residual endotoxin was removed from IgG preparations and from Ars-MSA by detergent extraction with Triton-X114 according to Aida and Pabst [67]. Endotoxin levels were determined by the *Limulus* amebocyte lysate test [68]. Endotoxin was undetectable in all samples except those used in the T_FH_ analysis, where it was less than 0.5 EU/sample. The DE52-purified IgG preparations were buffer exchanged from 10 mM NaPO4 pH 7.9 into a low aggregation pharmaceutical buffer (20 mM histidine, 222 mM trehalose dihydrate pH 5.5), adjusted to 4 mg/ml and passed through a 0.22 μm filter [69]. Polysorbate 80 (PS80) (Sigma Aldrich, St Louis, MO) was added to 0.02% (v/v) before freezing at -20°C in 500 μL aliquots. Individual tubes of IgG were subjected to a single freeze-thaw cycle prior to physical analyses or injection into animals.

### Generation of deaggregated, aggregated, and complexed IgG

To prepare the deaggregated form, frozen samples of IgG were thawed (once only/sample), diluted in sterile PBS to a concentration of 1 mg/ml and centrifuged at 165,000 x g in a fixed angle TLA-120.1 rotor (Beckman Coulter, Brea, CA) for 3 h. The top 2/3rds of the supernatant was removed and stored at 4°C for <6 hours prior to use. To prepare the heat-aggregated form, IgG (7 mg/ml) was buffer exchanged from low aggregation pharmaceutical buffer into endotoxin-free PBS. 1 ml aliquots were incubated at 63°C for 20 min. and immediately placed on ice for 1 h. Samples were centrifuged at 16,000 x g for 5 minutes, and the quantity of IgG remaining in the supernatant was determined (OD 280) in order to calculate the mass of the pelleted aggregated fraction, which was subsequently washed 3 times with low aggregation pharmaceutical buffer and stored at -20°C. Immune complexes were generated by incubating (3 h, 37°C, with rotation) 100 μg of mAb 36–71 with an amount of arsanilated mouse serum albumin (Ars-MSA, 13–15 haptens/MSA) four times greater than its mass at the equivalence point in a total volume 100 μl of PBS. Complexes were used within 2 hours of generation and never frozen or stored prior to use.

### Fab and F(ab’)_2_ generation with ficin

MAb 36–71 was digested using immobilized ficin (Thermo Fisher Scientific, Waltham, MA) according to the manufacturer’s protocol. The digested material was buffer exchanged into PBS and size excluded via fast protein liquid chromatography. Fab and F(ab’)2 fragments were buffer exchanged into the low aggregation pharmaceutical buffer, passed through a 0.22 μm filter, and stored at -20°C.

### Size-exclusion chromatography

Analytical size-exclusion chromatography was performed using an Agilent 1100 chromatography system (Agilent Technologies, Santa Clara, CA) as described [69, 70]. Prepared stocks of ultracentrifuged Ig, heat-aggregated Ig, immune complexes, or diluent PBS were spun at 13,000 x *g* and the supernatant removed to eliminate large insoluble particles. Protein was loaded onto a Tosoh G3000 SWXL 7.8 x 30 cm column (Tosoh Bioscience, Tokyo, Japan) and eluted with a mobile phase of PBS pH 7.4 at a flow rate of 1 ml/min. The eluate was monitored at 280 nm. Triplicate samples were analyzed for each Ig preparation.

### Microflow imaging

A Brightwell (Ottawa, ON, Canada) 4100 instrument was used for microflow imaging (MFI) to assess particle size and particle counts as described [69, 70]. 550 μl of Ig sample was loaded to allow total volume analysis of 500 μl; this analysis was performed in triplicate for each IgG preparation. The instrument was configured to allow for 1–50 μm particle detection by using “set point 3” mode and low magnification.

### Particle tracking analysis

A NanoSight LM20 (NanoSight Ltd., Amesbury, UK) instrument with a 405 nm laser was used for particle tracking analysis (PTA) as described [70]. 500 μL of sample was loaded into the flow chamber before data acquisition. Video was captured for 60 seconds using NTA 2.3 software at a setting recommended for low polydispersity samples, with size detection limit automatically determined by the software (1–600 nm), and with manually defined shutter and gain settings. Triplicate readings were obtained for each IgG preparation.

### Adoptive transfers and immunizations

After harvesting, a fraction of lymph node cells was stained and assayed by flow cytometry to identify percentages of CA30 T cells (CD4^+^Vβ8^+^). T cells were diluted with sterile PBS to a concentration of 5.0 x 10^5^/ml. For proliferation experiments, T cells were labeled with 5 mM CFSE prior to dilution. Mice received an i.v. transfer (100 μl) of cells and were rested for 24 hours prior to injection of IgG samples. The day of IgG injection was defined as “day 0”. Unless otherwise indicated, mice received primary antigen injections i.p. (100 μl sterile PBS) 24 hours after transfer of T cells. In some cases, antigen was precipitated in alum by mixing 1:1 (v/v) with 0.2 AlKSO4 followed by addition of 1M NaCO3. The precipitate was washed 3x in sterile PBS, and resuspended in sterile PBS for injection. For the anti-CD20 B cell depletion experiment, mice were injected i.p. with 500 μg of 5D2, an anti-mouse CD20 IgG2a/κ or control anti-ragweed monoclonal IgG2a/κ both generously donated by Genentech (San Francisco, CA) in sterile low aggregation pharmaceutical buffer 24 hours prior to i.v. adoptive transfer (day -2) of 5 x 10^4^ CFSE-labeled T cells and 48 h prior to antigen injection.

### Flow cytometry

Splenocytes were stained in the presence of α-CD16/32 as described [71]. The following antibodies were used: from eBioscience: α-CD8α (53–6.7) eFluor 450, α-CD45.1 (A20) APC, α-F4/80 (BM8) eFluor 450, α-CD19 (eBio1D3) eFluor 450, α-Vβ8.1/Vβ8.2 (KJ16-133) FITC, α-MHCII I-A/I-E (M5/114.15.2) eFluor 450, Rat IgG2a/κ isotype control (eBR2a) PE, α-BCL6 (BCL-DWN) PE, and α-CXCR5 (SPRCL5) Biotin; Biolegend: α-CD4 (GK1.5) APC-Cy7 and APC, α-B220/CD45R (RA3-6B2) FITC, PE, APC, APC-Cy7, eFluor 450, and α-PD-1 (RMP1-30) PE; Thermo Fisher: streptavidin-APC; Tonbo Biosciences: α-CD45.1 (A20) APC. Congenic CD4^+^ T cells were identified by first gating for forward and side scatter and then for CD4^+^, MHC II^-^, CD19^-^, F4/80^-^, CD8α^-^, at times using the latter gate to enrich for CD4^+^ events. Data were acquired on a FACScan, LSRII, or CyAn ADP flow cytometer and analyzed using FlowJo 9.7.1 (Tree Star, Ashland, OR). Cell numbers were calculated from splenocyte counts and cell percentages determined by flow cytometry.

### Serology

Serum IgG anti-Id was quantified using a target monoclonal IgM (ArsA11.1) that has the antigenic κ-light chain of mAb 36–71 [72, 73]. IgG binding to ELISA trays coated with ArsA11.1 was detected in a Eu^3+^-based flouroimmunometric assay using a biotinylated anti-IgG followed by streptavidin- Eu^3+^ as described [57]. Quantification was done using a standard curve generated with the IgG2b anti-Vκ^36–71^ mAb 17–63 [57]. Regression analysis was performed using Prism graphing software (GraphPad Software, La Jolla, CA).

### Statistics

Statistical analyses were performed using PRISM 5.0 (GraphPad, La Jolla, CA). Statistical analyses were made between samples using a two-tailed Mann-Whitney *U* test as specified in the figure captions.

## Results

### Aggregated and immune complexed mAb 36–71 elicit an IgG anti-Id response

We developed an adoptive transfer system to assess the response of CD4+ T cells to antigenic IgG in various physical states. A pair of IgG1 antibodies derived from hybridomas generated during an immune response to the hapten *p*-azophenylarsonate (Ars) served as experimental and control IgG antigens [66, 74, 75]. Both express the same VH/D/JH/Vκ/Jκ genes, but one, mAb 36–71, contains somatic mutations, including a pair in the Vκ framework-1 region that generate an antigenic I-A^k^-restricted antigenic peptide, referred to as pVκ^36–71^. The control IgG mAb 36–65 has no somatic mutations. Immunogenicity for CD4+ T cells was assessed with transgenic CA30 T cells, which express an αγ TCR that recognizes pVκ^36–71^ in the context of I-A^k^ [55]. The CA30 TCR Tg was maintained on an A/J genetic background and crossed with a CD45.1^+^ C57BL6 mouse to produce congenically marked CA30^+^ CD4 T cells for adoptive transfer.

**Fig 1:**
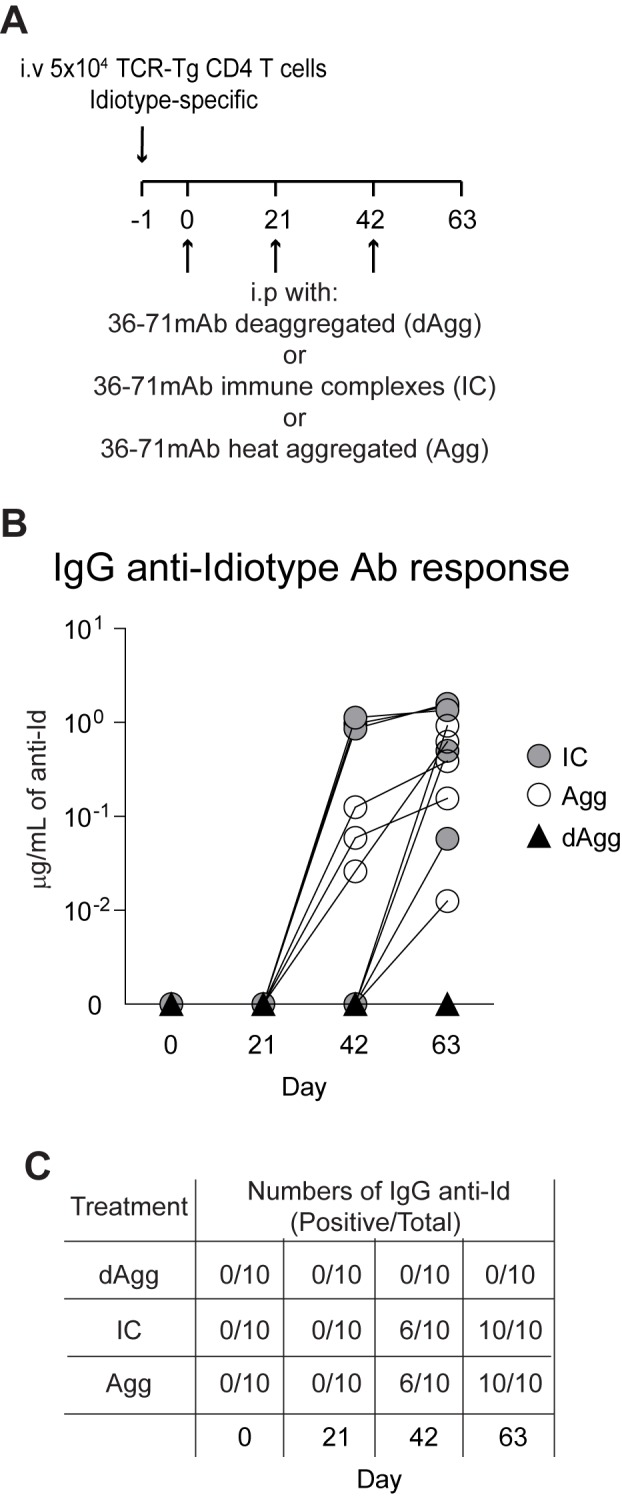
Heat-aggregated and immune complexes of mAb 36–71 elicit an IgG anti-Id response without requiring adjuvant. (A) Adoptive transfer and immunization scheme. (B) Serum IgG anti-idiotypic antibodies directed against the Vκ of mAb 36–71 were quantified using DELFIA as described in Materials and Methods. Connected lines denote a single mouse. Data are representative of two independent experiments with n = 5 mice per group. (C) Table summarizing results from 2 experiments with n = 5 mice per group with positive titers at each time point by treatment. Both experiments had 3 mice with positive titers at d42 and 5 mice with positive titers at d63 in both the heat-aggregated and immune complex groups.

To assess the potential immunogenicity of mAb 36–71 (IgG1), we transferred CA30 T cells (5 x 10^4^) into adoptive recipients, which were injected i.p. one day later (day 0) with deaggregated, heat-aggregated, or immune complexed mAb 36–71 (100 μg IgG in all cases). Mice were bled at day 21, at which time all groups were injected once again followed by a second bleed and boost at day 42 and final bleed at day 63 (Fig 1A). We then assayed sera for IgG anti-Id directed against the Vκ of mAb 36–71 (Fig 1B). None of the mice that received deaggregated mAb 36–71 developed detectable IgG anti-Id by day 63. In contrast, for the group that received heat-aggregated mAb 36–71, IgG anti-Id was detected in 3/5 mice at day 42 and in 5/5 mice by day 63 (Fig 1C). Similarly, in the group that received immune complexed mAb 36–71, IgG anti-Id was detected in 3/5 mice at day 42 and in 5/5 mice by day 63. Thus, endotoxin-free aggregated mAb 36–71 and IC mAb 36–71 were immunogenic, while deaggregated IgG was not.

### Deaggregated IgG induces antigen-specific tolerance in an Fc-dependent manner

**Fig 2:**
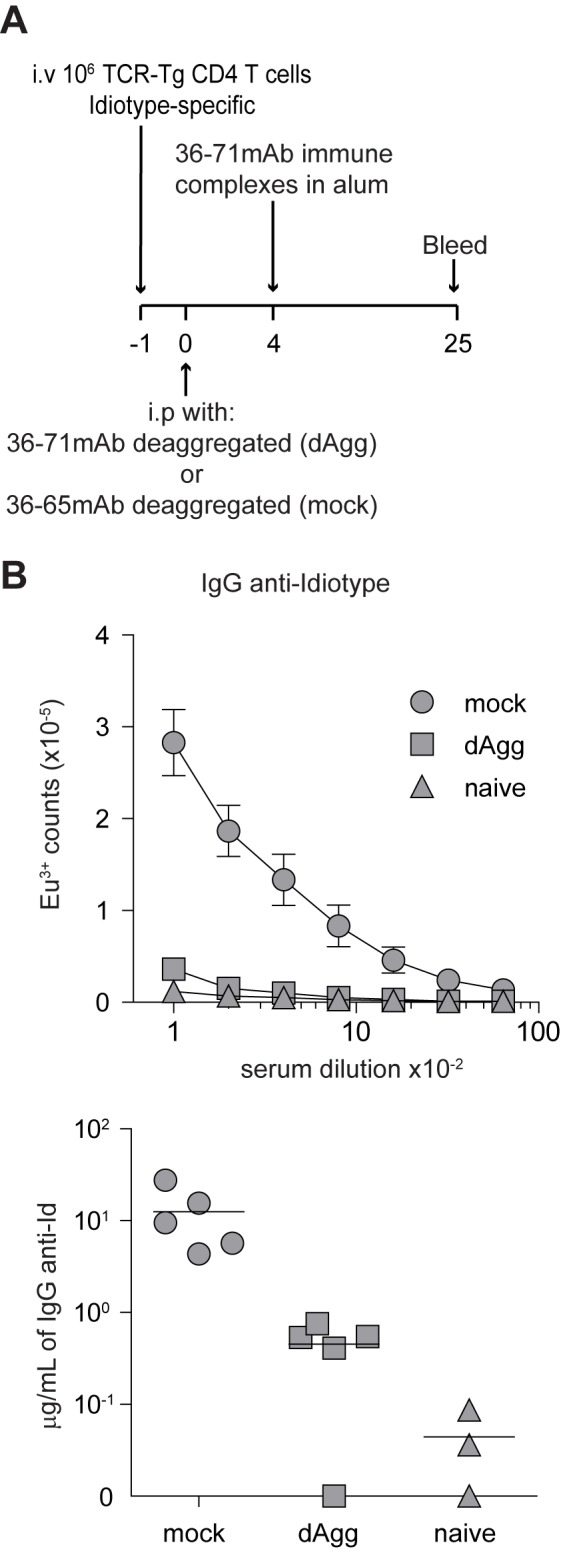
Deaggregated mAb 36–71 suppresses a humoral anti-Id response against mAb 36–71. (A) Adoptive transfer and immunization scheme. Primary and secondary injections were as specified in the figure. Naïve mice were B6AF1 mice that received CA30 cells, but no primary or secondary injection. (B) Mean titration curves (above) and concentrations (below) of serum IgG anti-Id at day 21. Each point represents an experimental mouse (n = 5/group). Results representative of 2 independent experiments with n = 5 mice per group.

Classic studies have revealed that monomeric, deaggregated IgG is tolerogenic with in the context of a wildtype CD4 T cell repertoire [5–10]. To determine if tolerance to IgG could be induced in an environment with an excess of potential T cell help, we injected mice with deaggregated mAb 36–71 i.p. twenty-four hours after adoptive transfer of 10^6^ CA30 T cells. Control animals received deaggregated mAb 36–65. Four days after these injections, the mice were immunized i.p.with IC mAb 36–71 in alum (Fig 2A). We added alum to provide a rigorous test of tolerance because in our hands alum usually increases the immune response to IC mAb 36–71 by more than 10-fold. Mice were bled at day 21 as in the previous experiment, and serum was tested for IgG anti-Id (Fig 2B). All of the mice that were initially treated with mAb 36–65 (mock) made IgG anti-Id responses. In contrast, treatment with deaggregated mAb 36–71 severely reduced the anti-Id responses in all mice (>30-fold). A repeat of this experiment with 5 x 10^4^ transferred CA30 T cells gave a similar result although less robust (~8-fold reduction, S1 Fig). Recipients in this experiment were younger (6–8 weeks old) than those of the first experiment (8–14 weeks old) and likely produced more thymic emigrants during the course of the experiment, which may have encountered the complexed form of mAb 36–71 before seeing the deaggregated form. These results show that, at the level of humoral immunity, deaggregated IgG induces self-tolerance, even in the presence of excess antigen-specific T cells.

### Aggregated and IC mAb 36–71 induce CA30 T cells to adopt a T follicular helper phenotype

**Fig 3:**
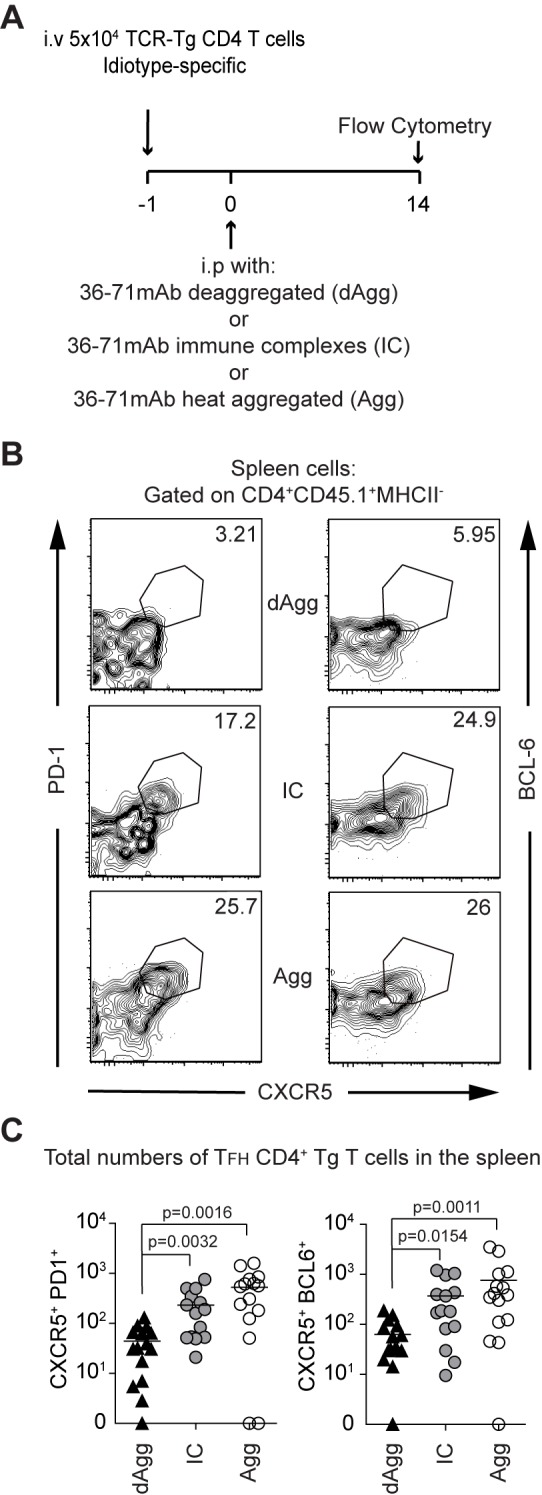
Heat-Aggregated and IC mAb 36–71 drive development of CA30 T_FH_ cells. (A) Adoptive transfer and immunization scheme. (B) Representative flow plots using the T_FH_ markers PD-1 and CXCR5 or Bcl-6 and CXCR5 respectively. Cells were pregated as CD45.1+, CD4+, MHC II-, CD19-, CD8α-, F4/80-. Numbers indicate percentages of CA30 T cells with indicated T_FH_ markers. (C) Numbers of CA30 T cells in individual mice with the indicated T_FH_ markers. Statistical differences were determined by a two-tailed Mann-Whitney *U* test. Data are representative of three independent experiments with n = 5 mice per group.

In view of the preceding results, we predicted that heat-aggregated and IC forms of mAb 36–71 would induce the development of T_FH_, while the deaggregated form would not. To test this, we performed our standard adoptive transfer and immunized mice with deaggregated, heat-aggregated or IC forms of mAb 36–71 and then stained at day 14 for splenic T_FH_ among CA30 T identified using the CD45.1 congenic marker (Fig 3A). While day 14 is expected to be within the contraction phase of the primary CD4+ T cell response, it is also at or near the peak of the germinal center reaction.

In mice that received aggregated mAb 36–71 or IC mAb 36–71, there were noticeable percentage increases in CA30 T cells that expressed the CXCR5^hi^PD-1^hi^ T_FH_ phenotype (Fig 3B). These increases were commensurate with significantly greater T_FH_ numbers (Fig 3C), which indicated an ~8-fold increase in CA30 T_FH_ cells in mice that received the heat-aggregated form of mAb 36–71 (p = 0.0016) and a 5.3-fold increase in those that had received the IC form (p = 0.0032) relative to the numbers in mice that received the deaggregated form. As the BCL-6 transcription repressor is critical in the development of T_FH_, we also confirmed increased percentages of CXCR5^hi^BCL6^+^ CA30 T cells in the groups that received heat-aggregated and IC mAb 36–71 relative to those that received the deaggregated form. In terms of CXCR5^hi^BCL6^+^ CA30 T cell numbers, the fold-increases in the heat-aggregated group and IC groups over those of the deaggregated group were nearly identical to those obtained using the PD-1^hi^ marker.

### All forms of mAb 36–71 induce efficient CA30 T cell proliferation, but aggregated and IC forms drive them through more division cycles

**Fig 4:**
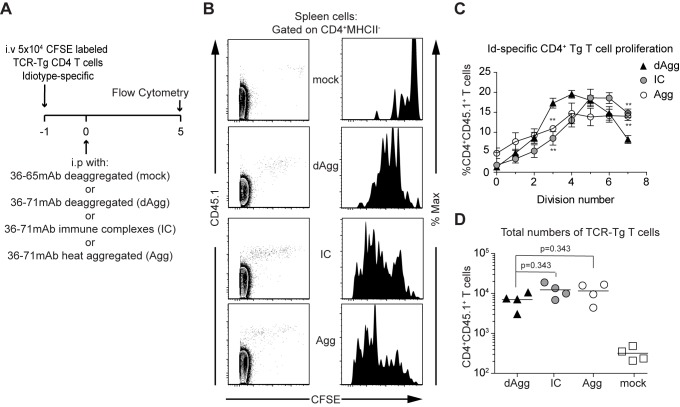
Aggregated and IC mAb 36–71 drive CA30 T cells through more division cycles than does the deaggregated form. (A) Adoptive transfer scheme. (B) Representative FACS plots of CFSE and CD45.1 staining in the CD4+, MHC II-, CD19-, CD8α-, F4/80- gate and representative CFSE histograms for CD4+ CD45.1+ (CA30) cells. (C) Graph of percentage or total numbers (D) of CD4+ CD45.1+ (CA30) cells with SEM in each cell cycle division gate as defined by the FlowJo CFSE proliferation algorithm. Data presented are from a single experiment with mice treated with deaggregated (n = 7), immune complex (n = 7), or heat aggregated (n = 4) mAb 36–71. Statistical differences were determined by a two-tailed Mann-Whitney *U* test (* = p<0.05, ** = p<0.005). Data are representative of 3 independent experiments using n ≥ 4 mice per group.

To examine the population dynamics of early CA30 T cell proliferation, we transferred CFSE-labeled CD45.1+ CA30 T cells into adoptive B6AF1 recipients and injected them with one of the 3 forms of mAb 36–71 or control mAb 36–65 the following day (Fig 4A). Five days after injection, splenocytes were assayed for CA30 T cells and CFSE profiles were examined. The congenic CD45.1^+^ marker allowed us to identify CA30 T cells that had divided rapidly and diluted the CFSE almost entirely. The resulting CA30 CFSE profiles revealed two notable trends (Fig 4B). First, deaggregated mAb 36–71 induced virtually all of the CA30 T cells to divide at least once, and a large percentage divided 3 to 5 times. Second, although all three forms of the IgG drove a high proportion of CA30 T cells to proliferate, the aggregated and IC forms drove more of the cells through 5 to 7 division cycles. To quantify this, we used the FlowJo proliferation algorithm to generate a set of gates for cell divisions, which were applied to each sample. This analysis showed that deaggregated mAb 36–71 drove most CA30 T cells to divide, with a majority falling between the 3^rd^ and 5^th^ division (Fig 4C and 4D). In contrast, the aggregated and IC forms drove the cells through more divisions, with the highest percentages of cells falling between the 5^th^ and 7^th^ division cycles.

### Removing the Fc from deaggregated mAb 36–71 abolishes the proliferative response by CA30 T cells

**Fig 5:**
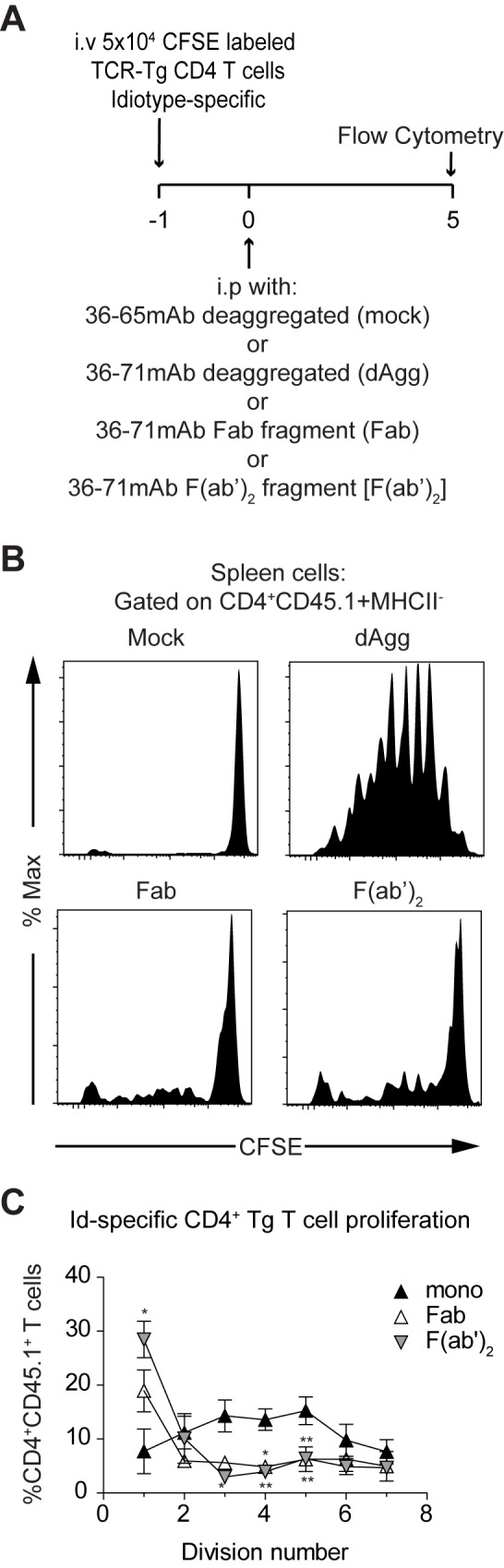
Removing the Fc from deaggregated mAb 36–71 ablates early proliferation of CA30 T cells. (A) Adoptive transfer and immunization protocol using deaggregated intact mAb 36–71 (100 μg) or the molar equivalent of its Fab (66 μg) or F(ab’)_2_ (73 μg) fragments, all deaggregated. (B) Representative CFSE histograms for CD4+ CD45.1+ (CA30) cells pregated in the CD4+, MHC II-, CD19-, CD8α-, F4/80- gate. (C) Graph of percentage of CD4+ CD45.1+ (CA30) cells with SEM in each cell cycle division gate as defined by the FlowJo CFSE proliferation algorithm. Data presented are from a single experiment with mice treated with deaggregated (n = 5), Fab (n = 5), or F(ab’)_2_ (n = 5) mAb 36–71. Statistical differences were determined by a two-tailed Mann-Whitney *U* test (* = p<0.05, ** = p<0.005). Data are representative of 2 independent experiments using n > 4 mice per group.

Given the numerous biological effector functions mediated by the antibody constant region, we sought to determine whether deaggregated mAb 36–71 lacking an Fc region was able to drive CD4 T cell proliferation as effectively as the intact form. To this end, we injected recipients of CFSE-labeled CA30 T cells with either mAb 36–65 (mock), intact deaggregated mAb 36–71, or its Fab or F(ab’)_2_ fragments and assessed T cell proliferation at day 5 (Fig 5A). In contrast to the intact mAb 36–71, both the Fab and F(ab’)_2_ fragments induced very little CA30 T cell proliferation (Fig 5B). As before, we validated and quantified this with the FlowJo proliferation algorithm, which confirmed that the monomeric Ig was driving most cells to divide, with a majority of the cells falling between the 3^rd^ and 5^th^ division (Fig 5C). In contrast, the F(ab’)_2_ and Fab induced few cells to divide beyond 1 or 2 divisions. Thus, the Fc component of Ig is needed for effective CD4 T cell proliferation *in vivo*.

### Physical analyses reveal marked size differences in subvisible particles between aggregated and IC mAb 36–71

Our experiments established that aggregated and complexed IgG were immunogenic for CD4+ T cells, while deaggregated monomeric IgG was not. Nevertheless, it was surprising that deaggregated mAb 36–71 was able to induce substantial proliferation by CA30 T cells (Figs 4 & 5), raising the possibility that some of it was aggregated. Therefore, we evaluated the physical states of the various preparations, using size-exclusion chromatography (SEC), particle-tracking analysis (PTA), and microflow imaging (MFI). SEC identifies soluble protein multimers (e.g. dimers, trimers). PTA identifies subvisible particles with radii in the nanometer range, and MFI identifies subvisible particles with radii in the micron range.

**Fig 6:**
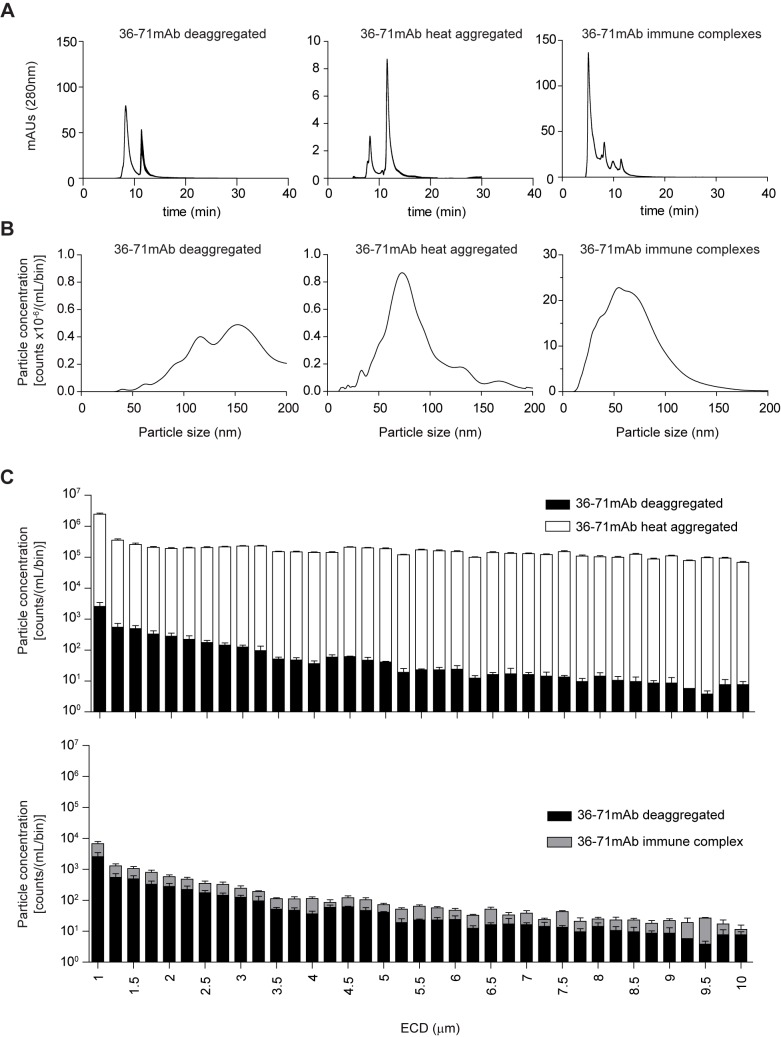
Heat-aggregated and IC mAb 36–71 are markedly different with respect to subvisible particle content. (A) Size-exclusion chromatograms comparing the 3 forms mAb 36–71 using milli-absorbance units (mAU). Note the change in Y-axis between heat aggregated and other samples (B) Particle concentration by size in nanometers determined by PTA. Note the change in Y-axis between immune complexes and other samples. (C) Particle concentration by size in microns determined by MFI defined by equivalent circular diameter (ECD). All analyses (SEC, PTA, MFI) were performed in triplicate on 3 biological replicates per sample, generated independently.

Size exclusion data for all three forms of mAb 36–71 are shown in Fig 6A. The chromatogram of the deaggregated preparation had a peak for monomeric IgG and another associated with the histidine-trehalose buffer at 8.14 and 13.1 minutes respectively. The aggregated preparation, on the other hand, contained only a minute amount of monomeric IgG. In fact, very little protein entered the SEC column once large insoluble particles were removed by a low speed centrifugation step (note the change in scale of the Y-axis). In contrast, a large amount of IgG entered the column from the IC preparation. This included some monomeric IgG, as well as complexed IgG, which was evident as a peak at 6.25 minutes. These size-exclusion data were consistent with PTA data, which revealed relatively few particles with radii in the 1 to 200 nm range (<10^6^ particles/ml/size bin) for the deaggregated and heat-aggregated preparations, each of which were within the range observed for the PBS/histidine-trehalose buffer controls (Fig 6B). In contrast, the IC preparation contained nanoparticles at concentrations that were >20-fold higher, particularly in the 50–75 nm range (note the change in scale of the Y-axis).

In the MFI analysis, we focused on subvisible particles with radii in the 1–10 **μ**m range, as these have previously been shown to be both the most prevalent subvisible particles in pharmaceutical mAbs and potentially immunogenic in animal models. There was 1000-fold more of these particles in the heat-aggregated preparation that in the deaggregated and IC preparations (Fig 6C).

To rule out the possibility that the observed proliferation induced by the deaggregated preparation of mAb 36–71 was due to trace levels of aggregated IgG, we immunized mice with IC or aggregated forms of mAb 36–71 that would be equivalent to the maximum possible contamination calculated from physical analyses performed on deaggregated mAb 36–71 using an integration of particle number and particle mass based on average protein density (1.43 g/ml) and particle diameter (excluding hydrated water) as described [69]. Accordingly, mice were immunized with either 5 μg of the IC form or 1 μg of the aggregated form of mAb 36–71 each diluted into 100 μg of deaggregated mAb 36–65 (S2A Fig). The latter served as a carrier to prevent loss of IC and aggregated mAb 36–71. When these were injected into recipients of CFSE-labeled CA30 T cells, virtually no proliferation was induced, as assessed by flow cytometric analyses at day 5 (S2B Fig).

### Excess local IgG diminishes antigen-specific CD4+ T cell proliferation against aggregated Ig, but not against immune complexes

**Fig 7:**
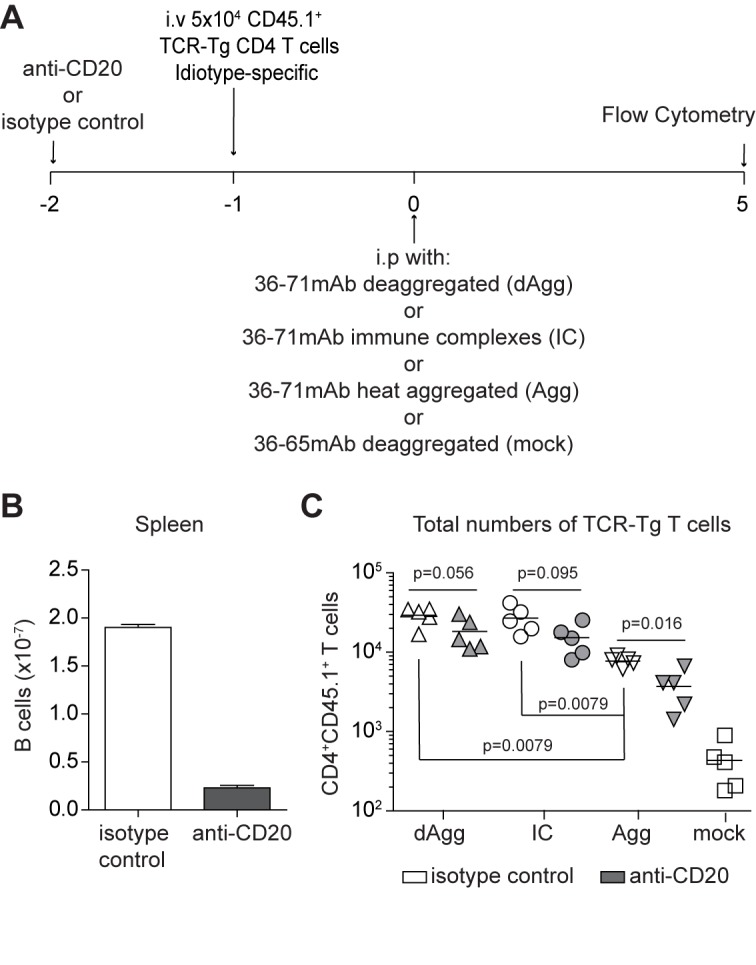
Aggregated mAb 36–71 requires B cells to induce early proliferation of CA30 T cells. (A) Adoptive transfer scheme designed to test early CA30 T cell proliferation after B cell depletion. (B) B220+ cell numbers at day 5. (C) CA30 T cell numbers at day 5. Data are representative of two independent experiments. Statistical differences were determined by a two-tailed Mann-Whitney *U* test with n = 5 mice per group. Data are representative of 2 independent experiments using n ≥ 5 mice per group.

In view of recent studies showing the influence of antigen size on its trafficking pattern in secondary lymphoid tissue, and the role of B cells as conveyors of particulate antigen to the germinal center, we speculated that B cells might be necessary for the CA30 T cell proliferation elicited by aggregates and IC of mAb 36–71. To test this, we injected 500 μg of anti-CD20 (5D2, IgG2a/κ) or a control IgG2a/κ (anti-ragweed) i.p. into mice one day prior to initiating our standard adoptive transfer with CA30 T cells (Fig 7A). Splenocytes were analyzed for B cell depletion and proliferation of the CA30 T cells 5 days after injection of deaggregated, heat-aggregated or IC mAb 36–71. The anti-CD20 treatment achieved a 78% reduction in B cells at this time (Fig 7B). When CA30 T cells were enumerated, there was a trend towards reduced numbers in all groups that were pretreated with the anti-CD20 mAb relative to those that received the control anti-ragweed mAb. However, this was most extreme in the mice that were immunized with heat-aggregated IgG, the only group in which the difference reached statistical significance (Fig 7C). Although it was not the intention of this experiment, the results also revealed that the control Ab resulted in diminished yields of CA30 T cells in the aggregate-immunized group. We can infer this because in all prior d5 proliferation experiments, the aggregate- and IC- immunized groups consistently yielded similar numbers of CA30 T cells and more so than the group immunized with deaggregated IgG (e.g. Fig 4D). In agreement with these results, CFSE profiles of CA30 cells in anti-CD20 and isotype control treatment groups were similar for mice immunized with either deaggregated IgG or IC and also similar to CFSE profiles in earlier experiments not involving anti-CD20. In contrast, CA30 T cells in mice immunized with heat aggregates had altered CFSE profiles relative to those of earlier experiments when treated with either anti-CD20 or isotype control IgG.

## Discussion

To investigate the dichotomy between the immunogenicity and tolerogenicity of IgG for CD4+ T cells, we developed an adoptive transfer and immunization protocol using αβTCR transgenic T cells (CA30) specific for a somatically mutated peptide within the Vκ region of the hapten-specific IgG, mAb 36–71. Our experiments demonstrated the immunogenicity of both aggregated and IC IgG under conditions in which no adjuvant was used and endotoxin contamination was nil. In stark contrast, we found that deaggregated IgG was highly tolerogenic, even when mice were challenged with immunogenic forms of IgG in a strong adjuvant and in the presence of excess T cell help. This contrast between immunogenicity and tolerogenicity was manifested by differences in CD4 T cell proliferation, differentiation into T_FH_ and production of a humoral IgG anti-Id response against the injected IgG.

Tolerogenicity of the deaggregated IgG was associated with efficient but attenuated proliferation of CD4+ CA30 T cells that was dependent upon the Fc region of the IgG. In contrast, induction of a humoral IgG anti-Id response by aggregated and IC IgG was associated with CD4+ T cell proliferation through a greater number of division cycles and T_FH_ development. Unexpectedly, CD4 T cell proliferation in response to the aggregated IgG was heavily dependent upon B cells but less so in response to IC IgG. These functional differences among the various forms of IgG were associated with differences in particulate matter composition. While the requirement of an Fc for effective T cell proliferation and tolerance could have several explanations, we think it is most likely due to the rapid clearance of Fab fragments *in vivo*, and the established role of the Fc in preserving serum Ab lifespan [76]. Consistent with this interpretation, we were unable to detect Fab in sera of mice injected only 24 h before (data not shown).

To our knowledge, these studies are the first to directly analyze how the physical state of IgG affects the activation of mAb-specific CD4+ T cells in an *in vivo* model of immune responses to mAb therapy. The observed immunogenicity of aggregated IgG concurs with both historical immunological and modern pharmaceutical science and justifies considerable efforts to eliminate these aggregates during production and handling. However, our results also indicate that removing denatured aggregates alone may not be sufficient to preclude immunogenicity because we found that IC were immunogenic. Thus, our data provide strong support for prior conjecture that complexes between therapeutic mAb and targeted antigen *in vivo* can be immunogenic and elicit IgG antibodies against the mAb [41–49]. This interpretation is consistent with our unexpected finding that B cell depletion strongly impedes a CD4 response against IgG aggregates but less so against IC because it suggests that anti-mAb responses by patients receiving B cell-depleting mAb, such as rituximab, may be due to complexes formed between the therapeutic mAb and its intended target.

Results of our B cell depletion experiments suggest that B cells in the peritoneum may be playing different roles in transport or presentation of aggregated IgG versus IC IgG. It has been reported that B cells are important for shuttling large antigens (dynamic radius >5.5 nm or ~70 kDa) into lymph node germinal centers, and more recent literature has substantiated this view by revealing their role in shuttling immune complexes [77–83]. However, in our model, CA30 T cell proliferation induced by IC was not as severely affected by B cell depletion as was proliferation induced by aggregated IgG. Perhaps this discrepancy is due to the small sizes of our IC compared to the aggregated mAb 36–71. Subvisible particle analyses revealed major differences between the heat-aggregated mAb 36–71 preparation, which had high concentrations of micron-sized particles, and IC mAb 36–71, which had high concentrations of nanometer-sized particles. We deliberately generated IC in antigen excess to favor smaller complexes based upon literature suggesting that smaller immune complexes are more inflammatory [84, 85]. Intriguingly, proliferation of CA30 T cells in the group immunized with heat-aggregated mAb 36–71 was somewhat diminished by pretreatment with the isotype control IgG. Although, IgG has been reported to impede other types of immune reactions, the mechanism by which it impedes T cell proliferative responses is unknown to us and would likely require extensive further investigation to uncover [76, 86, 87].

By necessity, a mAb must engage its target antigen for therapeutic effect, meaning that for many therapeutic mAb, immune complexes will be generated *in vivo* even if they were completely deaggregated during infusion. However, the efficacy of mAbs in the clinic depends upon their high affinity for antigen, which in turn is heavily dependent upon somatic diversity generated at V gene segment boundaries by terminal transferase and throughout the V region by somatic hypermutation. And this somatic diversity is a source of potential immunogenicity, even in fully human mAb. In our model for example, mAb 36–71 was originally derived from a strain A/J mouse. Yet somatic mutations rendered it antigenic with respect to the immune system of (B6 x A/J) F1 mice, and aggregation further rendered it immunogenic. Unfortunately, it is not a simple matter to selectively revert mutations that impart immunogenicity with respect to CD4 T cells because some mutations may both improve affinity and impart immunogenicity and because specific mutations that impart immunogenicity will likely vary with the MHC of the patient. These considerations lead us to conclude that in order to avert immune responses against therapeutic mAb it will often be necessary to induce a state of tolerance to the mAb in the CD4 T cell repertoire. In this vein, several groups have sought to suppress anti-mAb responses by injecting IgG with variable regions that were altered to prevent antigen engagement in vivo. [22, 24, 41, 49]. However, this strategy risks introducing an autoreactive specificity or eliminating an important T cell determinant to which tolerance is desired. So it is likely that novel tolerance strategies will have to be devised.

## Supporting Information

S1 Fig Deaggregated mAb 36–71 suppresses a humoral anti-Id response against mAb 36–71 in a mouse with a smaller CA30 T cell transfer. Mice 6–8 weeks of age received adoptive transfer of 5 x 10^4^ CA30 T cells on day -1 and 100 μg of deaggregated mAb 36–71 (experimental) or mAb 36–65 (control). On day 4, they received injection of 100 μg mAb 36–71 immune complexes in alum. Anti-idiotypic antibodies were measured on day 21. Mean titration curves of serum IgG anti-Id at day 21. Each point represents an experimental mouse (n = 5/group).
(EPS)

S2 Fig Trace levels of aggregated or immune complexed mAb 36–71 do not induce CA30 T cell proliferation. (A) Adoptive transfer and immunization scheme designed to test CA30 T cell proliferation. (B) Representative histograms showing CA30 T cell proliferation to 100 μg of immune complexed or aggregated mAb 36–71 (dotted) or 5 μg immune complexed mAb 36–71 + 100 μg control mAb 36–65 (shaded, left) or 1 μg aggregated mAb 36–71 + 100 μg control mAb 36–65 (shaded, right).
(EPS)

S3 Fig Representative CFSE profiles of CA30 T cell proliferation in B cell depletion experiments. Mice were pretreated with isotype control of anti-CD20 IgG (day -2), received adoptively transferred CA30 T cells (day -1) and species of mAb36-71 (day 0) as described in Fig 7A. Representative dot plots and histograms showing CA30 T cell proliferation to 100 μg of aggregated, immune complexed, or deaggregated mAb 36–71 on day 5. Percentages expressed are percentages of CD4+ CD45.1+ (CA30) cells pregated in the CD4+, MHC II-, CD19-, CD8α-, F4/80- gate.
(EPS)
